# Perfusion Territory Shifts in Cerebrovascular Diseases Measured by Super‐Selective Arterial Spin Labeling

**DOI:** 10.1111/jon.70101

**Published:** 2025-11-12

**Authors:** Gabriel Hoffmann, Miriam Reichert, Jens Göttler, Michael Helle, Lena Schmitzer, Moritz Hernandez Petzsche, Claus Zimmer, Christine Preibisch, Michael Kallmayer, Kornelia Kreiser, Nico Sollmann, Hans Liebl, Stephan Kaczmarz

**Affiliations:** ^1^ Department of Diagnostic and Interventional Neuroradiology, School of Medicine and Health Technical University of Munich (TUM) Munich Germany; ^2^ School of Medicine and Health TUM‐Neuroimaging Center, TUM Munich Germany; ^3^ Philips Innovative Technologies Hamburg Germany; ^4^ School of Medicine and Health Clinic of Neurology, TUM Munich Germany; ^5^ Department of Vascular and Endovascular Surgery, School of Medicine and Health TUM Munich Germany; ^6^ Department of Radiology and Neuroradiology Universitäts‐ und Rehabilitationskliniken Ulm Ulm Germany; ^7^ Department of Diagnostic and Interventional Radiology University Hospital Ulm Ulm Germany; ^8^ Philips GmbH Market DACH Hamburg Germany

**Keywords:** angiography, cerebrovascular diseases, magnetic resonance imaging, super‐selective arterial spin labeling, vascular territories

## Abstract

**Background and Purpose:**

Individualized diagnostic approaches are crucial in cerebrovascular diseases, such as internal carotid artery stenosis (ICAS). To evaluate individual collateral blood supply, vessel‐selective imaging has gained high relevance. However, clinically established digital subtraction angiography (DSA) exposes patients to intervention risks and radiation. Two noninvasive MRI‐based alternatives are super‐selective pseudo‐continuous arterial spin labeling (ss‐pCASL, a technique for selective labeling of arterial blood‐water) for perfusion territory mapping and four‐dimensional vessel‐selective angiography (4D‐sPACK). We hypothesized that asymptomatic atherosclerosis‐induced ICAS and Moyamoya disease result in chronic malperfusion. Therefore, we aimed towards quantitative assessment of collateral blood flow by ss‐pCASL.

**Methods:**

In this prospective monocentric study, we acquired data in three subgroups (*n* = 23): patients with asymptomatic unilateral atherosclerosis‐induced ICAS, Moyamoya disease, and age‐matched healthy controls (HCs). On the basis of vascular territories from ss‐pCASL, we introduced four parameters: volume, territorial shift, overlap with an atlas, and cerebral blood flow (CBF). For patients with atherosclerosis‐induced ICAS, ipsi‐ and contralateral hemispheres were compared (paired *t*‐test), and hemispheric lateralization *Δ* was calculated subjectwise and compared between patients and HCs (unpaired *t*‐test) (*p *< 0.05).

**Results:**

We included data from 20 subjects (8 ICAS, 3 Moyamoya, 9 HC). Group‐level results showed ICAS‐induced shifts with significant lateralization compared to HCs (*Δ*_Volume,ICAS_ = 18% ± 10%, *p *< 0.001; *Δ*_Shift,ICAS_ = 4.9% ± 5.8%, *p* = 0.027; *Δ*_Overlap,ICAS_ = 0.2 ± 0.3, *p* = 0.033, *Δ*_CBF,ICAS_ = 3 ± 3 mL/100 g/min, *p* = 0.045). Furthermore, collateral blood supply in Moyamoya disease was assessed by 4D‐sPACK and showed comparable diagnostic value as DSA.

**Conclusion:**

Perfusion territory mapping by ss‐pCASL revealed chronic malperfusion in asymptomatic ICAS that can be objectively quantified, and 4D‐sPACK added diagnostic value similar to DSA.

## 1 Introduction

Cerebrovascular diseases (CVD) are a significant health issue and are associated with an increased risk for ischemic stroke [1, 2]. A major representative of CVD is internal carotid artery stenosis (ICAS), which is usually seen in elderly subjects with cardiovascular risk factors, resulting in atherosclerosis‐induced ICAS, as well as in rare diseases such as Moyamoya disease, which commonly manifests in younger patients [2, 3, 4, 5]. Although CVD typically results in chronic hypoperfusion of the dependent territory of the brain due to the narrowing of the supplying vessels, there are protective mechanisms such as collateral blood flow [6, 7]. Collateralization heavily depends on individual anatomical configuration regarding the Circle of Willis and individual disease‐related pathophysiological changes of vascular structure. Altogether, this leads to a high demand for noninvasive imaging of individual vascular morphology as well as perfusion territories, temporal dynamics, and related perfusion characteristics. To date, clinically available catheter‐based digital subtraction angiography (DSA) depicts the individual vessels’ anatomy and blood supply at high resolution; however, it is an invasive procedure that comes with intervention risks and radiation exposure [8].

A viable noninvasive alternative to DSA may be super‐selective pseudo‐continuous arterial spin labeling (ss‐pCASL) that can be obtained by MRI without any application of intravenous contrast agents [9]. Instead, it uses the blood water as an endogenous tracer by magnetically labeling spins within a defined region of the brain‐feeding arteries [9, 10]. Previous studies have shown the feasibility of ss‐pCASL for (qualitative) perfusion territory mapping, especially in CVD [11, 12]. Although ss‐pCASL allows for vessel‐specific perfusion territory mapping, selective labeling can also be extended to four‐dimensional (4D) magnetic resonance angiography (MRA) using additional contrast‐enhanced timing‐robust angiography (CENTRA)‐keyhole and view‐sharing 4D vessel‐selective angiography (4D‐sPACK) [13, 14]. Thereby, dynamic time‐resolved angiograms can be obtained from a single vessel, depicting blood inflow into individual vascular territories. Recent work has shown its applicability to a wide range of pathologies, such as arterio‐venous malformations [15, 16] or Moyamoya disease [17].

Although previous work on ss‐pCASL focused on qualitative descriptions of perfusion alterations [11, 12, 17, 18], the purpose of this study was to evaluate the potential of quantitative perfusion territory mapping by ss‐pCASL with combined 4D‐sPACK for CVD, specifically in patients with ICAS related to atherosclerosis or Moyamoya disease. We hypothesized that (asymptomatic) ICAS induces shifts of perfusion territories, which can be measured using ss‐pCASL. We further hypothesized that the methodology may also be used for detecting perfusion territory shifts in Moyamoya disease and, in combination with 4D‐sPACK, gives diagnostic value comparable to DSA noninvasively.

## 2 Methods

The local institutional review board approved this prospective monocentric study. All participants provided written informed consent.

### 2.1 Subjects

Our study cohort included 23 subjects within three subgroups: 11 patients with unilateral asymptomatic ICAS from atherosclerosis, 3 patients with unilateral ICAS from Moyamoya disease, and, with respect to ICAS, 9 age‐matched healthy controls (HCs). The inclusion criterion for patients was stenosis degree >70% according to the North American Symptomatic Carotid Endarterectomy Trial (NASCET) criterion [19], which was determined by duplex ultrasonography. Additionally, vascular anatomical variants of the anterior circulation that may simulate disease, such as fetal norm variants of the Circle of Willis, were ruled out based on time‐of‐flight (TOF)‐MRA. Exclusion criteria were any neurological, psychiatric, or systemic diseases; clinically relevant structural MRI lesions (such as territorial stroke, bleedings, or a history of brain surgery); severe chronic kidney disease; and general MRI contraindications. A final sample size of 20 subjects (8 patients with atherosclerosis‐induced ICAS, age 69.8 ± 6.2 years, 5 female; 3 patients with Moyamoya disease, age 31.7 ± 3.8 years, 3 female; 9 HCs, age 68.2 ± 5.8, 6 female) was included, as three patients did not meet the inclusion criteria.

### 2.2 MRI Acquisition

**FIGURE 1:**
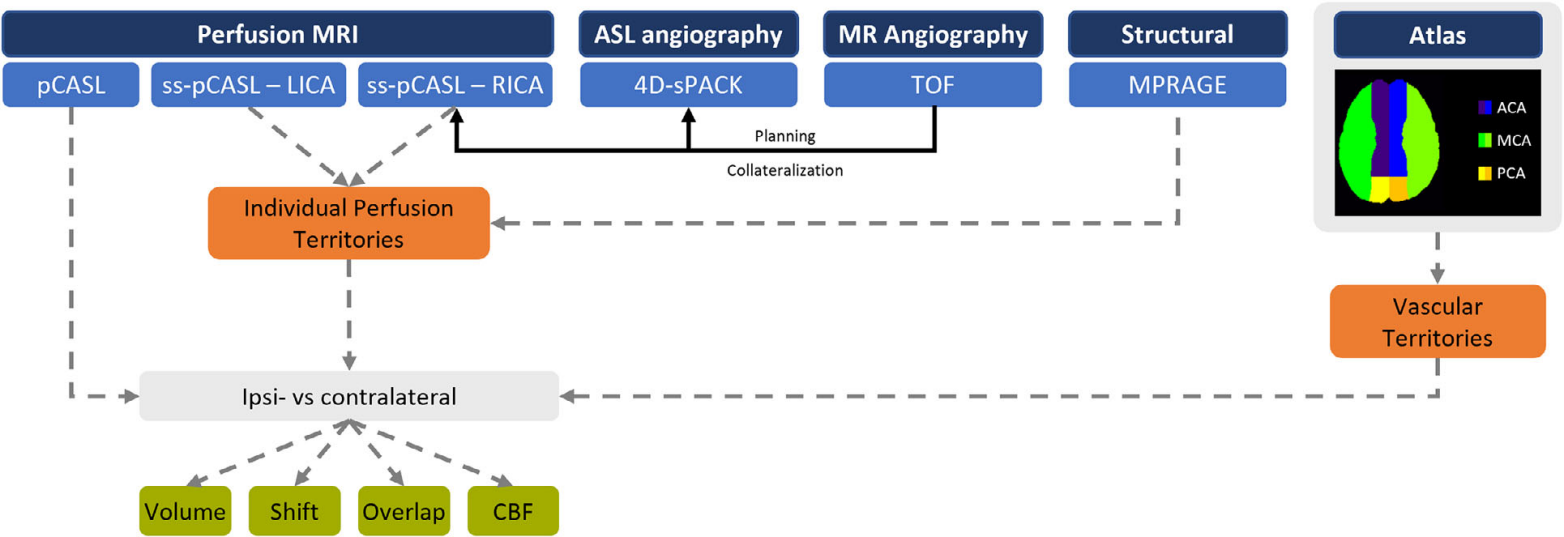
MRI protocol and derived parameters. Perfusion MRI included whole‐brain pseudo‐continuous arterial spin labeling (pCASL) yielding quantitative cerebral blood flow (CBF) maps, as well as super‐selective ASL (ss‐pCASL) derived from separate labeling of the left and right internal carotid arteries (LICA and RICA). ASL‐angiography was performed by means of 4D‐MR angiography (4D‐sPACK). Time‐of‐flight (TOF) angiography was used for assessing both the individual subject's vessel architecture and collateralization status, as well as for planning of the ss‐pCASL and 4D‐sPACK sequences. Vessel‐specific perfusion territories were compared between hemispheres and to an MNI‐based atlas of vascular territories of the anterior, middle, and posterior cerebral arteries (ACA, MCA, PCA) [24]. MPRAGE, magnetization prepared rapid gradient echo.

Image acquisition was performed on 3T MRI scanners (Ingenia Elition X or Achieva Quasar Dual; Philips Healthcare, Best, the Netherlands) using 32‐channel head coils (*n* = 12) or 20‐channel (*n* = 11) head‐neck coils. A multi‐sequence MRI protocol was applied (Figure 1). Specifically, TOF‐MRA was used for individual planning of the ss‐pCASL label positions as well as to assess the subjects’ intracranial vessel status with respect to exclusion criteria. Importantly, the imaging protocol included two different pCASL sequences: an ss‐pCASL sequence for perfusion territory mapping and a nonselective pCASL sequence for perfusion quantification as cerebral blood flow (CBF). The additional pCASL was needed given that ss‐pCASL is commonly not considered to be quantitative due to varying labeling efficiencies in individual vessels [20].

pCASL was applied for whole‐brain CBF and implemented according to current recommendations [10, 21], with a label duration (LD) of 1800 ms and a post‐label delay (PLD) of 1800 ms for HCs and 2000 ms for patients, respectively. Different readout schemes were applied according to availability on the respective system: segmented three‐dimensional (3D) gradient and spin echo (GraSE) readout (echo time TE = 7.4 ms, repetition time TR = 4403 ms, 16 slices, turbo spin echo factor 19, echo planar imaging (EPI) factor 7, acquired voxel size 2.75 × 2.75 × 6 mm^3^, 3 dynamics, 5:43 min) was used in 19 subjects, and two‐dimensional (2D) EPI (TE = 11 ms, TR = 4500 ms, 20 slices, EPI factor 29, acquired voxel size 3.3 × 3.5 × 6 mm^3^, 25 dynamics, 4:02 min) in 4 subjects. Background suppression was applied. Proton density‐weighted (PDw) M_0_ scans were additionally acquired to quantify CBF.

TOF‐MRA covered at least the volume between the carotid bifurcation and the terminal branches of the intracranial large vessels (i.e., A3 and M3 segments of the anterior cerebral artery [ACA] and middle cerebral artery [MCA]) in vertical dimension, thus also covering the Circle of Willis, using a 3D GraSE readout (TE = 3.5 ms, TR = 25 ms, *α* = 20°, SENSE factor 2, acquisition voxel size 1.5 × 1.5 × 1.5 mm^3^, 1:43 min).

V*essel‐selective imaging* included both ss‐pCASL [9] for perfusion territory mapping, as well as 4D‐sPACK [14, 16] for noninvasive, time‐resolved, and super‐selective MRA. The left and right ICAs were labeled separately. The location of the labeling spot was determined on the basis of the vessel's location in TOF images. Labeling and geometric parameters were mostly identical to nonselective pCASL; however, they were slightly accelerated using parallel imaging for 3D readout (SENSE = 1.8) and using 23 instead of 25 dynamics for 2D readout, resulting in scan times of approximately 3 min both for 3D and 2D readouts per labeled vessel. Ss‐pCASL and 4D‐sPACK were acquired separately. Functionalities were provided by the ASL extensions patch (Philips Healthcare, Best, the Netherlands).

Structural imaging comprised a *T*1‐weighted magnetization prepared rapid gradient echo (MPRAGE) sequence (TE = 4 ms, TR = 9 ms, inversion time (TI) = 757 ms, *α* = 8°, acquisition voxel size = 1 × 1 × 1 mm^3^, 5:59 min).

### 2.3 Image Processing

Identical image processing was performed for the patient groups and HCs using custom‐built MATLAB programs (version R2021b, The MathWorks Inc., Natick, MA, USA) and statistical parametric mapping (SPM 12, Wellcome Trust Centre for Neuroimaging, UCL, London, UK). Structural *T*1‐weighted imaging data were segmented into white matter (WM) and grey matter (GM) tissue probability maps, and *p* > 0.7 was used to generate tissue masks. Although for ss‐pCASL, qualitative perfusion‐weighted images were directly used for analyses, pCASL data were used for CBF quantification according to Alsop et al. [10]. The CBF maps were scaled as previously published [22, 23]. Moreover, CBF values in GM and WM were extracted using the *T*1‐based segmentation.

### 2.4 Quantitative Analyses of Perfusion Territories

An identical two‐step analysis was performed for the analysis of perfusion territory shifts in all three groups. First, masks of vascular perfusion territories were derived from vessel‐selective ss‐pCASL perfusion maps from each labeled ICA. This was done by semi‐automatic segmentation (performed by three readers) using Vinci software (version 5.06, Max‐Plank‐Institute for Neurological Research, Cologne, Germany). Ss‐pCASL perfusion maps were opened as an overlay on co‐registered anatomical T1w data. Then an adequate threshold was defined by the reader that allowed for delineation of the respective perfusion territory, and any obvious artifacts were removed (e.g., rim intensities due to imperfect motion correction, apparent signal outside the brain). Second, four quantitative parameters were calculated to individually characterize the blood supply from each ICA (Figure 1):

1. fractional volume,
2. territorial shift,
3. overlap an atlas of vascular territories [24],
4. CBF.

**FIGURE 2:**
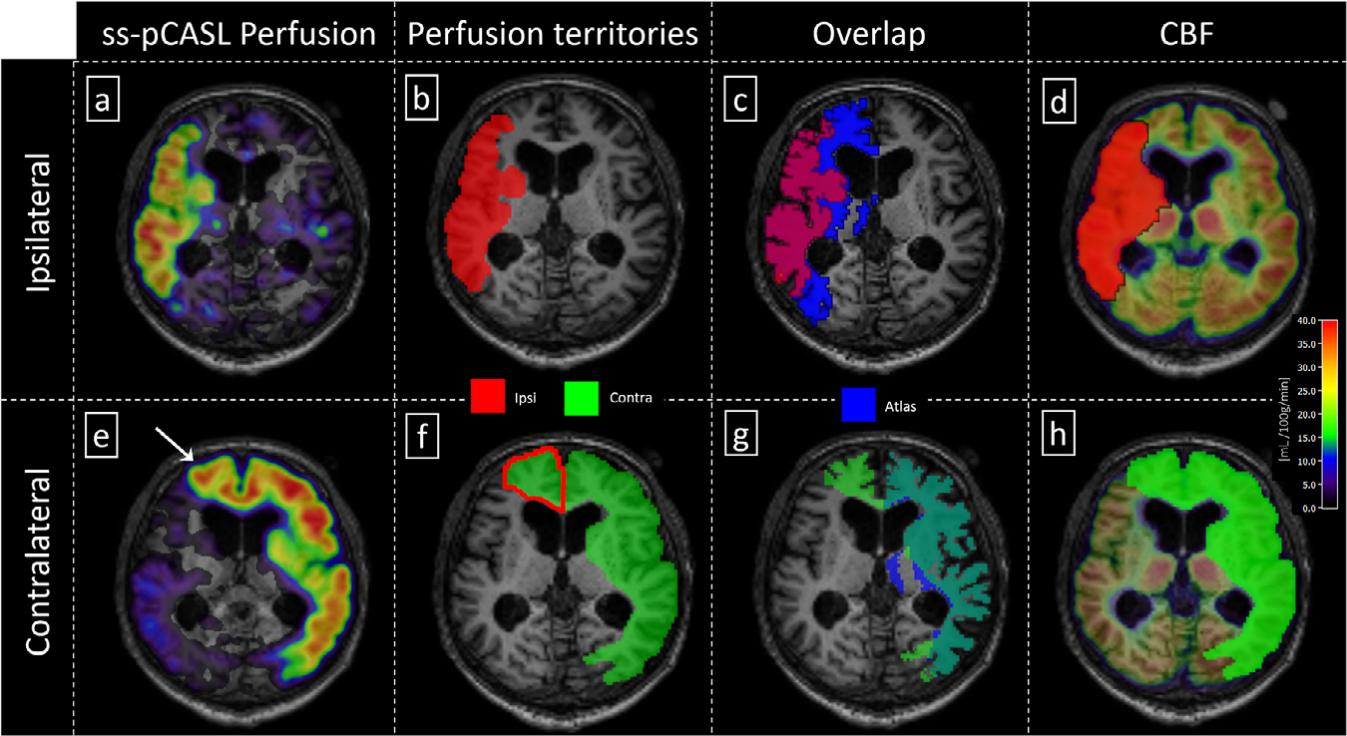
Exemplary patient (right‐sided stenosis, 77 years, male). Ipsilateral (a–d) and contralateral (e–h) hemispheres are shown for the same axial slice. Segmentation of super‐selective pCASL (ss‐pCASL) perfusion maps (a and e) yielded vessel‐selective perfusion territory masks of the ipsi‐ (b, red) and contralateral (f, green) hemispheres. Perfusion territories of the non‐stenosed internal carotid artery were notably larger with compensatory contralateral supply (f). The fractional shift of the contralateral territory is indicated by a red frame. Atlas‐based vascular territories of the anterior circulation are shown in blue and were compared to vessel‐selective individual vascular territory masks with additional grey and white matter masks applied (c and g), yielding more extensive overlap for the contralateral carotid artery. For cerebral blood flow (CBF), nonselective, quantitative pCASL‐based CBF maps were evaluated within vessel‐selective territories (d and h).

The fractional volume of each vascular territory, supplied by a selectively labeled ICA, was calculated from the segmented volume of the territory in comparison to the acquired whole‐brain volume, calculated from perfusion maps. The shift of a perfusion territory was defined as the fractional volume of a hemisphere that was perfused by the opposite ICA (Figure 2). The overlap of single‐vessel perfusion territories with a standardized vessel configuration was assessed by co‐registering the 3D atlas of arterial vascular territories from Liu et al. [24] onto the individual subjects’ vessel‐selective ss‐pCASL perfusion maps. Registration used spm_coreg from SPM 12 (rigid body transformation) using default parameters. Subsequently, DICE coefficients of spatial overlaps were calculated (DICE = 1 indicates complete congruence of vascular territory masks from ss‐pCASL and the atlas, DICE = 0 signifies no overlap). Lastly, absolute CBF within individual vascular territories was calculated by evaluating the nonselective pCASL‐based CBF maps within region of interests defined by the vessel‐selective territory maps from ss‐pCASL of each ICA.

For group‐level visualization, we defined a standard side for stenotic disease and mirrored imaging data accordingly, if needed. Individual perfusion territories were normalized to Montreal Neurological Institute (MNI) standard space, which allowed the calculation of group mean probability maps of territories. In the following, the terms ipsi‐ and contralateral will be used with respect to the side of the stenosis.

### 2.5 Visual Image Assessment

In addition to quantitative analyses of perfusion territories, two readers (over 5 years of experience in neuroradiology each) evaluated the 4D‐sPACK data in the three patients with Moyamoya disease, together with the DSA data (available in two of those cases). Images were opened and rated using the image viewer of the local Picture Archiving and Communication System (PACS; IDS7, Sectra AB, Linköping, Sweden). The rating scheme from 0 to 5 is based on a previously published approach for assessment of TOF‐MRA images (0 = vessel not depictable, 5 = no artifacts, no compromise of diagnostic quality) [25, 26].

### 2.6 Statistical Analyses

Group‐level statistical analyses were performed for patients with atherosclerosis‐induced ICAS and HCs cohort using MATLAB. For each of the above‐defined four parameters, we tested for normal distribution using Shapiro–Wilk and Anderson–Darling tests, assuming that *p* > 0.05 indicates the normal distribution of the respective parameter values. Both tests indicated normal distribution for all parameters, but the territorial shift. Accordingly, we used paired *t*‐tests to compare average values for hemispheres ipsi‐ and contralateral to the stenosis for normally distributed values and Kolmogorov–Smirnov tests for the shift. In addition, we calculated for each parameter (*P*) its lateralization (*∆*_P_):

(1) $$\Delta_{P}=P_{\mathrm{contra}/\mathrm{left}}-P_{\mathrm{ipsi}/\mathrm{right}}\;P\in \left\{\mathrm{sh}\mathrm{ift};\mathrm{volume};\mathrm{CBF};\mathrm{overlap}\right\}$$

Lateralization of the ICAS patients was compared against HC using non‐paired *t*‐tests or Wilcoxon rank sum tests, respectively. Results are given as mean values with errors indicating standard deviation. A *p* value <0.05 was considered statistically significant.

## 3 Results

### 3.1 Patients With Atherosclerosis‐Induced ICAS

Exemplary data of a patient with right‐sided ICAS showed a marked shift in perfusion territories (Figure 2). Brain regions in the anterior circulation ipsilateral to the stenosis (Figure 2a) were fed by the non‐stenosed contralateral ICA (Figure 2e). The segmentation of vessel‐selective perfusion territories showed that the non‐stenosed ICA perfused a clearly larger volume (26% of the hemisphere, Figure 2f) than the stenosed vessel (11%, Figure 2b). Accordingly, the contralateral perfusion territory perfused 1.9% fractional volume of the ipsilateral hemisphere, indicated by the red frame (Figure 2f). Moreover, the overlap of the individual perfusion territory with the atlas was larger for the contralateral hemisphere (Figure 2g, DICE = 0.7) compared to ipsilateral regions (Figure 2c, DICE = 0.6). Finally, CBF was calculated by evaluating global perfusion maps from pCASL within the vessel‐selective perfusion territories from ss‐pCASL, yielding CBF values of 38 mL/100 g/min contralateral (Figure 2h) and 34 mL/100 g/min ipsilateral to the stenosis, respectively (Figure 2d).

**FIGURE 3:**
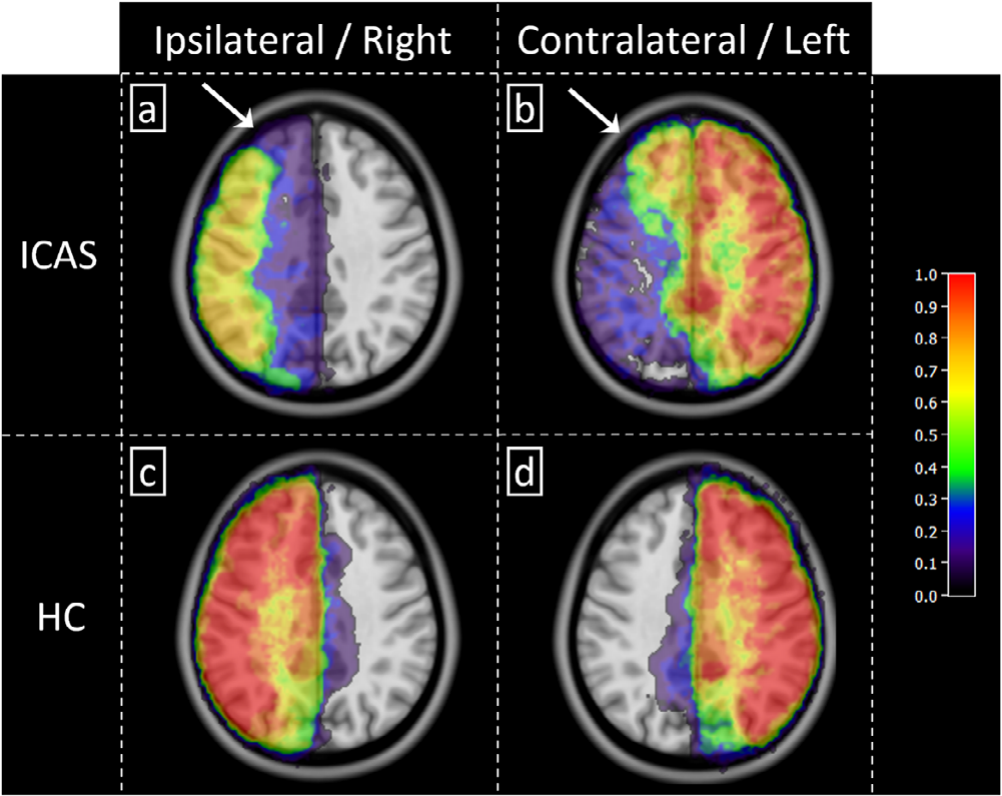
Group analysis. Probability maps of mean vascular territories indicated strong shifts of perfusion territories induced by ICAS from atherosclerosis, with compensatory crossflow from the respective contralateral carotid artery (b) towards hypoperfused regions of the ipsilateral hemisphere (a, arrows). Compared to that, healthy controls’ (HC) perfusion territories remained symmetric (c and d). ICAS, internal carotid artery stenosis.

Probability maps of group mean vascular territories (Figure 3) demonstrated shifts in the anterior circulation. On average, perfusion territories ipsilateral to the stenosed ICA were markedly smaller (Figure 3a) and partially supplied from the contralateral circulation (Figure 3b). In contrast to the apparent shifts in patients, the perfusion territories of HCs were widely symmetric among hemispheres (Figure 3c,d).

**FIGURE 4:**
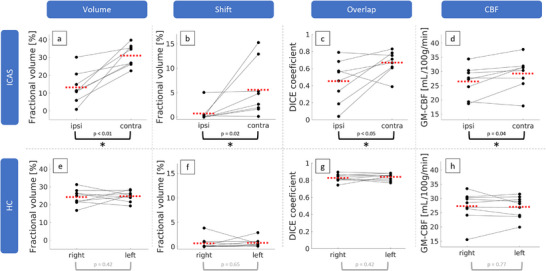
Paired scatterplots evaluating hemispheric differences between individual perfusion territories for atherosclerosis‐induced internal carotid artery stenosis (ICAS) patients (a–d) and healthy controls (HCs, e–h). For ICAS patients, the fractional volume perfused by the non‐stenosed carotid artery (contra) was significantly larger than the fractional volume perfused by the stenosed ICA (ipsi) (a). Accordingly, ICAS patients showed significantly larger hemispherical shifts from the contralateral to the ipsilateral side (b). In addition, there was significantly larger overlap with atlas‐based territories (c) and elevated cerebral blood flow (CBF) (d) contralateral to the stenosis. Compared to that, HC perfusion territories were symmetrical in all four parameters (e–h). Dots represent mean values of each subject. Red dashed lines indicate group mean values. Asterisks indicate statistically significant differences between hemispheres (p < 0.05)

Statistical evaluations comparing contra‐ and ipsilateral parameters (Figure 4 and Table 1) yielded significantly larger partial volumes perfused by the contralateral ICA (31% ± 6% vs. 13% ± 9%; Figure 4a). Correspondingly, contralateral perfusion territories were significantly shifted to the ipsilateral hemisphere by a fractional volume of 5.9% ± 5.6% (Figure 4b). We found significantly higher overlap of the vessel‐selective territories with the atlas for contralateral hemispheres (DICE_contra_ = 0.7 ± 0.1 vs. DICE_ipsi_ = 0.5 ± 0.3, Figure 4c) as well as ipsilaterally decreased CBF values (26 ± 5 mL/100 g/min vs. 29 ± 6 mL/100 g/min, Figure 4d). In the HC group, all parameters were symmetrical on group level, with some variability across single subjects (Figure 4e–h).

**TABLE 1:** Quantitative parameters for (atherosclerosis‐induced) internal carotid artery stenosis (ICAS) and Moyamoya patients and healthy controls (HCs) for hemispheres ipsi‐ and contralateral to the stenosis, or right and left, respectively.

	Volume (%)			Shift (%)			Overlap			CBF (mL/100 g/min)		
	Ipsi/r	Contra/l	*∆*_Volume_	Ipsi/r	Contra/l	*∆*_Shift_	Ipsi/r	Contra/l	*Δ*_Overlap_	Ipsi/r	Contra/l	*Δ*_CBF_
ICAS	13 ± 9	31 ± 6	18 ± 10	0.7 ± 1.8	5.6 ± 5.6	4.9 ± 5.8	0.5 ± 0.3	0.7 ± 0.1	0.2 ± 0.3	26 ± 5	29 ± 5.8	3 ± 3
Moyamoya	29 ± 6	38 ± 5	9 ± 10	2.4 ± 1.9	7.2 ± 4.7	4.8 ± 3.8	0.8 ± 0.1	0.8 ± 0.1	0.0 ± 0.0	28 ± 2	30 ± 2	2 ± 2
HC	24 ± 4	25 ± 29	0 ± 5	0.7 ± 1.2	0.8 ± 0.9	0.1 ± 1.5	0.8 ± 0.0	0.8 ± 0.0	0.0 ± 0.1	27 ± 5	27 ± 4	0 ± 3

*Note*: Mean values of volume, shift, overlap, and cerebral blood flow (CBF) are given with errors indicating standard deviation.

Abbreviations: CBF, cerebral blood flow; l, left; r, right.

**FIGURE 5:**
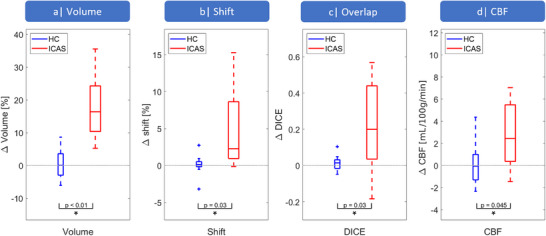
Whisker‐Boxplots comparing the lateralization *∆* between atherosclerosis‐induced ICAS patients (shown in red) and healthy controls (HC, blue). For ICAS we found significantly increased *∆* volume (a), *∆* shift (b), *∆* overlap (c), and *∆* cerebral blood flow (CBF, d). Whisker plot boxes indicate 25th and 75th percentiles, median values are shown with a horizontal line, and outliers are visualized by crosses. Asterisks indicate statistically significant differences between patients and HCs (*p *< 0.05). ICAS, internal carotid artery stenosis.

The lateralization *∆* (Equation 1) was found to be significantly larger for each of the parameters in patients compared to HCs (Figure 5, Table 1). In detail, we found lateralization in volume (*Δ*_Volume,ICAS_ = 18% ± 10%, Figure 5a), shift (*Δ*_Shift,ICAS_ = 4.9% ± 5.8%, Figure 5b), overlap (*Δ*_Overlap,ICAS_ = 0.2 ± 0.3, Figure 5c), and CBF (*Δ*_CBF,ICAS_ = 3 ± 3 mL/100 g/min, Figure 5d) for patients, whereas HCs had *∆* ≈ 0 for all parameters.

### 3.2 Patients With Moyamoya Disease

**FIGURE 6:**
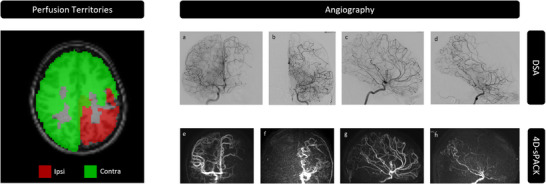
Exemplary data of Moyamoya patient 1. Perfusion territories are shown in an axial slice both ipsilateral (red) and contralateral (green) to the side of the Moyamoya‐induced stenoses (left). Angiography (right) is compared between digital subtraction angiography (DSA, a–d) and 4D‐sPACK (four‐dimensional vessel‐selective angiography) (e–h). Coronal DSA indicated collateral supply towards the ipsilateral hemisphere (a and c) in concordance with reduced blood supply of the stenotic ICA (b and d). Similarly, noninvasive time‐resolved 4D‐sPACK showed collateralization for the non‐stenotic internal carotid artery (e and g), whereas the ipsilateral hemisphere is mal‐perfused (f and h).

Exemplary imaging data of one patient with Moyamoya disease and a near‐total stenosis of the left distal ICA and the left MCA are shown in Figure 6. Specifically, ss‐pCASL‐based vessel‐selective perfusion territories indicated marked collateral blood supply from the right contralateral ICA towards the left hemisphere (Figure 6, left). This concurred with vessel‐selective angiography (Figure 6, right), where noninvasive 4D‐sPACK (Figure 6e–h) resembled DSA findings in coronal and sagittal views (Figure 6a–d). The strong hypoperfusion of the ipsilateral hemisphere, which was due to the MCA stenosis (Figure 6b,f and d,h), was compensated by pronounced compensatory blood supply with strong crossflow from the right ICA via the right A1 segment and anterior communicating artery to the enlarged left ACA (Figure 6a,e, and c,g). Quantitative analyses yielded a substantial territorial shift of 11.4% and a difference of 20% in perfused volume. Similarly, higher contralateral CBF (*Δ*_CBF_ = 3.87 mL/100 g/min) and increased territorial overlap (*∆*_DICE_ = 0.1) were found.

The results of the two other patients with Moyamoya disease compared in general well with the other patient with Moyamoya disease and patients with ICAS due to atherosclerosis (Table 1).

According to visual image assessment with a standardized rating scheme, the intraosseous and intradural part were clearly depicted without artifacts by 4D‐sPACK data as well as DSA (score: 5). Furthermore, the ACA and MCA were visualized by 4D‐sPACK data as well as DSA regarding their A1/M1, A2/M2, and A3/M3 segments without compromises (score: 3). The ophthalmic artery was discernible in all cases as well (score: 1). The pathology in the sense of a stenosis of the M1 segment of the MCA could be delineated by both 4D‐sPACK as well as DSA images. Ratings of the other two patients also indicated similar accuracy for 4D‐sPACK (Table 2).

**TABLE 2:** Consensus rating (0 = vessel not depictable, 5 = no artifacts, no compromise of diagnostic quality) for all included cases with Moyamoya‐induced stenosis (no DSA available for Patient 2).

	DSA						4D‐sPACK					
Patient 1	0	1	2	3	4	5	0	1	2	3	4	5
												
Intraosseous ICA						×						×
Intradural ICA						×						×
ACA				×						×		
MCA				×						×		
Ophthalmic artery		×						×				
												
Patient 2	0	1	2	3	4	5	0	1	2	3	4	5
												
Intraosseous ICA	n.a.	n.a.	n.a.	n.a.	n.a.	n.a.						×
Intradural ICA	n.a.	n.a.	n.a.	n.a.	n.a.	n.a.						×
ACA	n.a.	n.a.	n.a.	n.a.	n.a.	n.a.				×		
MCA	n.a.	n.a.	n.a.	n.a.	n.a.	n.a.				×		
Ophthalmic artery	n.a.	n.a.	n.a.	n.a.	n.a.	n.a.		×				
												
Patient 3	0	1	2	3	4	5	0	1	2	3	4	5
												
Intraosseous ICA						×						×
Intradural ICA						×						×
ACA				×						×		
MCA				×						×		
Ophthalmic artery		×						×				

*Note*: Consensus rating (0 = vessel not depictable, 5 = no artifacts, no compromise of diagnostic quality) for all included cases with Moyamoya‐induced stenosis (no digital subtraction angiography (DSA) available for Patient 2 = n.a.).

Abbreviations: 4D‐sPACK, four‐dimensional vessel‐selective angiography; ACA, anterior cerebral artery; ICA, internal carotid artery; MCA, middle cerebral artery; n.a., not available.

## 4 Discussion

In this study, we demonstrated that ss‐pCASL allows for the assessment of individual perfusion territories and CVD‐induced changes in blood supply. We introduced four quantitative parameters that revealed considerable lateralization differences between patients with ICAS and HCs. As hypothesized, both investigated CVD entities (atherosclerosis‐induced ICAS and Moyamoya disease) introduced measurable collateral blood supply towards hypoperfused regions.

Our results indicate that asymptomatic ICAS from atherosclerosis induces shifts in vascular territories, which may be quantified by ss‐pCASL‐based perfusion territory segmentation. This is noteworthy given the mixed findings in the literature: although some studies reported perfusion territory shifts for highly stenosed and symptomatic patients [18, 27], other studies found insignificant shifts for asymptomatic patients with lower degrees of stenosis [28]. In our study, we evaluated the perfusion status of the anterior circulation, namely, the ACA and MCA territories, by separate labeling of the left and right ICA. Group mean vascular territory maps (Figure 3) showed distinct shifts from contralateral hemispheres for ICAS patients, which clearly contrasted with symmetrical perfusion patterns of the HCs. Compared to other studies based mainly on visual inspection of selective perfusion maps [12, 29], we substantiated those findings by quantitative topographical analyses (Figures 4 and 5).

The group mean territory maps, together with hemispheric shifts and asymmetric overlaps, may indicate collateral blood supply, altering the location of vascular territories. Consecutively, shifts of the border zones occur at the junction of the perfusion territories. Given that those border zones are predominantly affected in CVD patients due to their remote localization [30, 31], identifying their precise locations is of particular interest for interpreting ischemic patterns. Furthermore, increased collateral blood supply towards ipsilateral hemispheres, as indicated by the lateralization *Δ*, agrees with already described shifts of vascular border zones in a similar cohort [32, 33]. This can be explained by reduced cerebral perfusion pressure in hemodynamically impaired regions. It also matches very well with a recent study [27], which showed, using another vessel‐selective ASL technique, that high‐grade ICAS induces asymmetry in perfusion territories. Furthermore, this previous study showed that the deviation of territories is more prevalent in higher degrees of stenosis [27]. This may also explain the results of another study, showing no territory shifts in ICAS when including asymptomatic patients with ICAS of lower grades (NASCET > 50%) [28]. Due to the exclusion of fetal norm variants of the Circle of Willis, we assume blood supply towards hypoperfused regions of the stenosed ICA, as reported in other studies [34, 35]. Additionally, lateralized CBF matched well with previously reported unilaterally lowered CBF in a similar cohort [22, 23].

All three patients with Moyamoya disease showed notable shifts in perfusion territories as well as different‐sized perfusion territory volumes. Both findings agree well with previous DSA‐based studies, reporting severe Moyamoya‐related heterogeneity in perfusion patterns and also illustrating a high degree of collateralization as a main characteristic of this pathology [36, 37, 38]. It also agrees with a recent publication, which compared ss‐pCASL to DSA [39]. Similarly, regional effects have been described in blood oxygen level dependent (BOLD) MRI and ASL reactivity studies [36, 38] or after revascularization therapy [40]. However, most previous studies either relied on nonselective MR perfusion measures [38] or invasive DSA [40]. We showed on the basis of visual impression and a quantitative rating that noninvasive 4D‐sPACK was in excellent accordance with DSA, indicating collateral blood supply towards MCA segments.

With respect to both investigated CVD entities, our results match well with previous studies, which have shown excellent applicability of ss‐pCASL for imaging cerebrovascular pathophysiology [12, 41]. The proposed methodology may have high potential for clinical diagnosis due to the easily applicable ss‐pCASL sequence as well as clinically relevant perspectives towards treatment decisions based on the individual quantification of perfusion alterations. In addition, ss‐pCASL and 4D‐sPACK allow a precise and flexible labeling of the investigated vessels, distinguishing it from other vessel‐selective methods such as territorial ASL [18, 42].

We have to consider the following limitations regarding our present study. Although ASL is widely available, it is not applicable after stenotic stenting due to susceptibility artifacts and SAR limitations in the neck region, as the ASL labeling plane needs to be placed here. Moreover, ss‐pCASL is not yet available on most clinical scanners as a product sequence but needs a research‐based setting. Even though ss‐pCASL sequences are not quantitative, and co‐registration of ss‐pCASL onto CBF involves an additional processing step, we are confident that our approach of evaluating nonselective pCASL within individual territories approximates the hemodynamic status adequately. Using this procedure implies that CBF values may also contain other sources of flow than just from the artery labeled by ss‐pCASL. A more rigorous approach would be to determine perfusion fraction instead of CBF [43]. Although this gives perfusion with flow sources only from a single labeled vessel, it needs additional information to correct for variations in labeling efficiency, which is usually not available in routine clinical scans. Moreover, CBF might be impacted by transit times, which we partially accounted for by using a prolonged PLD. Additionally, we calculated spatial covariance values below 0.45, indicating the absence of severe transit time artifacts as proposed by Mutsaerts et al. [44]. Nevertheless, especially for Moyamoya patients, CBF might still be slightly biased as ATT even substantially beyond 2000 ms has already been reported [45]. Lastly, we have only a limited number of Moyamoya cases included in our study.

In this study, we showed the potential of MRI‐based ss‐pCASL and 4D‐sPACK for investigating CVD‐induced perfusion alterations. By introducing quantitative parameters based on noninvasive ss‐pCASL, we could show that asymptomatic ICAS induces shifts in perfusion territories. In Moyamoya disease, those parameters resembled angiography findings. Therefore, noninvasive 4D‐sPACK showed similar image information to that of DSA.

## Funding

This study received funding from Ev. Studienwerk Villigst (personal grant to GH), Deutsche Forschungsgemeinschaft (DFG, German Research Foundation)—Projektnummer 395030489 (grant to C.P.) and Projektnummer 547163214 (grant to S.K. and J.G.) and Dr.‐Ing. Leonhard Lorenz‐Stiftung (grant to S.K. 971/19).

## Disclosure

Parts of this work have already been presented as conference abstract at the 2022 Joint Annual Meeting ISMRM‐ESMRMB & ISMRT 31st Annual Meeting in London, UK (2022) and at the BRAIN & BRAIN PET 2022, the 30th International Symposium on Cerebral Blood Flow, Metabolism, and Function in Glasgow, UK.

## Conflicts of Interest

The authors declare no conflicts of interest.

## References

[1] M. J. Donahue , E. Achten , P. M. Cogswell , et al., “Consensus Statement on Current and Emerging Methods for the Diagnosis and Evaluation of Cerebrovascular Disease,” Journal of Cerebral Blood Flow and Metabolism 38 (2018): 1391–1417. 28816594 10.1177/0271678X17721830 PMC6125970

[2] G. W. Petty , R. D. Brown Jr. , and J. P. Whisnant , “Ischemic Stroke Subtypes: A Population‐Based Study of Incidence and Risk Factors,” Stroke; A Journal of Cerebral Circulation 30 (1999): 2513–2516. 10.1161/01.str.30.12.2513 10582970

[3] M. L. Flaherty , B. Kissela , J. C. Khoury , et al., “Carotid Artery Stenosis as a Cause of Stroke,” Neuroepidemiology 40 (2012): 36–41. 23075828 10.1159/000341410 PMC3626492

[4] C. L. Hallemeier , K. M. Rich , R. L. Grubb Jr. , et al., “Clinical Features and Outcome in North American Adults With Moyamoya Phenomenon,” Stroke; A Journal of Cerebral Circulation 37 (2006): 1490–1496. 10.1161/01.STR.0000221787.70503.ca 16645133

[5] M. Kraemer , W. Heienbrok , and P. Berlit , “Moyamoya Disease in Europeans,” Stroke; A Journal of Cerebral Circulation 39 (2008): 3193–3200. 10.1161/STROKEAHA.107.513408 18787200

[6] D. Liebeskind , “Collateral Circulation,” Stroke; A Journal of Cerebral Circulation 34 (2003): 2279–2284. 10.1161/01.STR.0000086465.41263.06 12881609

[7] S. Jung , R. Wiest , J. Gralla , et al., “Relevance of the Cerebral Collateral Circulation in Ischaemic Stroke: Time Is Brain, but Collaterals Set the Pace,” Swiss Medical Weekly 147 (2017): w14538. 29231236 10.4414/smw.2017.14538

[8] R. A. Willinsky , S. M. Taylor , K. terBrugge , et al., “Neurologic Complications of Cerebral Angiography: Prospective Analysis of 2,899 Procedures and Review of the Literature,” Radiology 227 (2003): 522–528. 12637677 10.1148/radiol.2272012071

[9] M. Helle , D. G. Norris , S. Rufer , et al., “Superselective Pseudocontinuous Arterial Spin Labeling,” Magnetic Resonance in Medicine 64 (2010): 777–786. 20597127 10.1002/mrm.22451

[10] D. C. Alsop , J. A. Detre , X. Golay , et al., “Recommended Implementation of Arterial Spin‐Labeled Perfusion MRI for Clinical Applications: A Consensus of the ISMRM Perfusion Study Group and the European Consortium for ASL in Dementia,” Magnetic Resonance in Medicine 73 (2015): 102–116. 24715426 10.1002/mrm.25197 PMC4190138

[11] M. Helle , S. Rufer , M. J. van Osch , et al., “Superselective Arterial Spin Labeling Applied for Flow Territory Mapping in Various Cerebrovascular Diseases,” Journal of Magnetic Resonance Imaging 38 (2013): 496–503. 23526786 10.1002/jmri.24041

[12] V. Richter , M. Helle , M. J. van Osch , et al., “MR Imaging of Individual Perfusion Reorganization Using Superselective Pseudocontinuous Arterial Spin‐Labeling in Patients With Complex Extracranial Steno‐Occlusive Disease,” AJNR American Journal of Neuroradiology 38 (2017): 703–711. 28183839 10.3174/ajnr.A5090 PMC7960227

[13] M. Obara , O. Togao , G. M. Beck , et al., “Non‐Contrast Enhanced 4D Intracranial MR Angiography Based on Pseudo‐Continuous Arterial Spin Labeling With the Keyhole and View‐Sharing Technique,” Magnetic Resonance in Medicine 80 (2018): 719–725. 29369424 10.1002/mrm.27074

[14] M. Obara , O. Togao , M. Helle , et al., “Improved Selective Visualization of Internal and External Carotid Artery in 4D‐MR Angiography Based on Super‐Selective Pseudo‐Continuous Arterial Spin Labeling Combined With CENTRA‐Keyhole and View‐Sharing (4D‐S‐PACK),” Magnetic Resonance Imaging 73 (2020): 15–22. 32763367 10.1016/j.mri.2020.07.013

[15] M. R. Hernandez Petzsche , M. Reichert , G. Hoffmann , et al., “Non‐Invasive Perfusion Territory Quantification and Time‐Resolved Angiography by Arterial Spin Labeling in a Patient With a Large Right‐Hemispheric AVM: Case Report,” Journal of Neurology 269 (2022): 1–7. 35279740 10.1007/s00415-022-11065-3 PMC9293865

[16] O. Togao , M. Obara , M. Helle , et al., “Vessel‐Selective 4D‐MR Angiography Using Super‐Selective Pseudo‐Continuous Arterial Spin Labeling May be a Useful Tool for Assessing Brain AVM Hemodynamics,” European Radiology 30 (2020): 6452–6463. 32696254 10.1007/s00330-020-07057-4

[17] N. Sollmann , H. Liebl , C. Preibisch , et al., “Super‐Selective ASL and 4D ASL‐Based MR Angiography in a Patient With Moyamoya Disease,” Clinical Neuroradiology 31 (2021): 515–519. 32975610 10.1007/s00062-020-00961-8 PMC8211600

[18] P. J. van Laar , J. Hendrikse , C. J. Klijn , et al., “Symptomatic Carotid Artery Occlusion: Flow Territories of Major Brain‐Feeding Arteries,” Radiology 242 (2007): 526–534. 17255422 10.1148/radiol.2422060179

[19] North American Symptomatic Carotid Endarterectomy Trial (NASCET) Steering Committee , “North American Symptomatic Carotid Endarterectomy Trial. Methods, Patient Characteristics, and Progress,” Stroke; A Journal of Cerebral Circulation 22 (1991): 711–720. 10.1161/01.str.22.6.711 2057968

[20] T. Lindner , O. Jansen , and M. Helle , “Optimized Super‐Selective Arterial Spin Labeling for Quantitative Flow Territory Mapping,” Magnetic Resonance Imaging 53 (2018): 14–19. 29966693 10.1016/j.mri.2018.06.020

[21] T. Lindner , D. S. Bolar , E. Achten , et al., “Current State and Guidance on Arterial Spin Labeling Perfusion MRI in Clinical Neuroimaging,” Magnetic Resonance in Medicine 89 (2023): 2024–2047. 36695294 10.1002/mrm.29572 PMC10914350

[22] J. Göttler , S. Kaczmarz , R. Nuttall , et al., “The Stronger One‐Sided Relative Hypoperfusion, the More Pronounced Ipsilateral Spatial Attentional Bias in Patients With Asymptomatic Carotid Stenosis,” Journal of Cerebral Blood Flow and Metabolism 40 (2020): 314–327. 30480463 10.1177/0271678X18815790 PMC7370612

[23] S. Kaczmarz , J. Göttler , J. Petr , et al., “Hemodynamic Impairments Within Individual Watershed Areas in Asymptomatic Carotid Artery Stenosis by Multimodal MRI,” Journal of Cerebral Blood Flow and Metabolism 41 (2021): 380–396. 32237952 10.1177/0271678X20912364 PMC7812517

[24] C.‐F. Liu , J. Hsu , X. Xu , et al., “Digital 3D Brain MRI Arterial Territories Atlas,” Scientific Data 10 (2023): 74. 36739282 10.1038/s41597-022-01923-0 PMC9899211

[25] T. Greve , N. Sollmann , A. Hock , et al., “Highly Accelerated Time‐of‐Flight Magnetic Resonance Angiography Using Spiral Imaging Improves Conspicuity of Intracranial Arterial Branches While Reducing Scan Time,” European Radiology 30 (2020): 855–865. 31664504 10.1007/s00330-019-06442-y

[26] T. Greve , N. Sollmann , A. Hock , C. Zimmer , and J. S. Kirschke , “Novel Ultrafast Spiral Head MR Angiography Compared to Standard MR and CT Angiography,” Journal of Neuroimaging 31 (2021): 45–56. 33118692 10.1111/jon.12791

[27] Y.‐F. Chen , S.‐C. Tang , W.‐C. Wu , et al., “Alterations of Cerebral Perfusion in Asymptomatic Internal Carotid Artery Steno‐Occlusive Disease,” Scientific Reports 7 (2017): 1841. 28500300 10.1038/s41598-017-02094-4 PMC5431826

[28] N. S. Hartkamp , E. T. Petersen , M. A. Chappell , et al., “Relationship Between Haemodynamic Impairment and Collateral Blood Flow in Carotid Artery Disease,” Journal of Cerebral Blood Flow and Metabolism 38 (2018): 2021–2032. 28776469 10.1177/0271678X17724027 PMC6238174

[29] P. J. van Laar , J. Hendrikse , X. Golay , et al., “In Vivo Flow Territory Mapping of Major Brain Feeding Arteries,” Neuroimage 29 (2006): 136–144. 16095923 10.1016/j.neuroimage.2005.07.011

[30] C. D'Amore and M. Paciaroni , “Border‐Zone and Watershed Infarctions,” in Manifestations of Stroke, ed. M. Paciaroni , G. Agnelli , V. Caso , J. Bogousslavsky (Karger Publishers, 2012), 181–184. 10.1159/000333638 22377891

[31] I. Momjian‐Mayor and J.‐C. Baron , “The Pathophysiology of Watershed Infarction in Internal Carotid Artery Disease: Review of Cerebral Perfusion Studies,” Stroke; A Journal of Cerebral Circulation 36 (2005): 567–577. 10.1161/01.STR.0000155727.82242.e1 15692123

[32] S. Kaczmarz , V. Griese , C. Preibisch , et al., “Increased Variability of Watershed Areas in Patients With High‐Grade Carotid Stenosis,” Neuroradiology 60 (2018): 311–323. 29299616 10.1007/s00234-017-1970-4

[33] L. Schmitzer , N. Sollmann , J. Kufer , et al., “Decreasing Spatial Variability of Individual Watershed Areas by Revascularization Therapy in Patients With High‐Grade Carotid Artery Stenosis,” Journal of Magnetic Resonance Imaging 54 (2021): 1878–1889. 34145686 10.1002/jmri.27788

[34] M. Kluytmans , J. van der Grond , K. J. van Everdingen , et al., “Cerebral Hemodynamics in Relation to Patterns of Collateral Flow,” Stroke; A Journal of Cerebral Circulation 30 (1999): 1432–1439. 10.1161/01.str.30.7.1432 10390319

[35] W. J. Powers , G. A. Press , R. L. Grubb Jr. , M. Gado , and M. E. Raichle , “The Effect of Hemodynamically Significant Carotid Artery Disease on the Hemodynamic Status of the Cerebral Circulation,” Annals of Internal Medicine 106 (1987): 27–34. 3491558 10.7326/0003-4819-106-1-27

[36] G. Zaharchuk , H. M. Do , M. P. Marks , et al., “Arterial Spin‐Labeling MRI Can Identify the Presence and Intensity of Collateral Perfusion in Patients With Moyamoya Disease,” Stroke; A Journal of Cerebral Circulation 42 (2011): 2485–2491. 10.1161/STROKEAHA.111.616466 PMC3164217 21799169

[37] G. A. Schubert , M. Czabanka , M. Seiz , et al., “Perfusion Characteristics of Moyamoya Disease,” Stroke; A Journal of Cerebral Circulation 45 (2014): 101–106. 10.1161/STROKEAHA.113.003370 24193795

[38] M. J. Donahue , M. Ayad , R. Moore , et al., “Relationships Between Hypercarbic Reactivity, Cerebral Blood Flow, and Arterial Circulation Times in Patients With Moyamoya Disease,” Journal of Magnetic Resonance Imaging 38 (2013): 1129–1139. 23440909 10.1002/jmri.24070 PMC3675170

[39] J. Yuan , J. Qu , Z. Lv , et al., “Assessment of Blood Supply of the External Carotid Artery in Moyamoya Disease Using Super‐Selective Pseudo‐Continuous Arterial Spin Labeling Technique,” European Radiology 31 (2021): 9287–9295. 34021389 10.1007/s00330-021-07893-y

[40] S. K. Lee , D. I. Kim , E. K. Jeong , et al., “Postoperative Evaluation of Moyamoya Disease With Perfusion‐Weighted MR Imaging: Initial Experience,” AJNR American Journal of Neuroradiology 24 (2003): 741–747. 12695215 PMC8148687

[41] S. Kaczmarz , M. Reichert , M. Hernandez‐Petzsche , et al., “Clinical Application of ASL‐Based Non‐Invasive Perfusion Territory Mapping and Time‐Resolved Angiography in Cerebrovascular Diseases,” in Proceedings of the International Society of Magnetic Resonance in Medicine , (2021).

[42] N. S. Hartkamp , M. Helle , M. A. Chappell , et al., “Validation of Planning‐Free Vessel‐Encoded Pseudo‐Continuous Arterial Spin Labeling MR Imaging as Territorial‐ASL Strategy by Comparison to Super‐Selective p‐CASL MRI,” Magnetic Resonance in Medicine 71 (2014): 2059–2070. 23878062 10.1002/mrm.24872

[43] J. Schollenberger , C. A. Figueroa , J. F. Nielsen , and L. Hernandez‐Garcia , “Practical Considerations for Territorial Perfusion Mapping in the Cerebral Circulation Using Super‐Selective Pseudo‐Continuous Arterial Spin Labeling,” Magnetic Resonance in Medicine 83 (2020): 492–504. 31418475 10.1002/mrm.27936 PMC7428084

[44] H. J. Mutsaerts , J. Petr , L. Václavů , et al., “The Spatial Coefficient of Variation in Arterial Spin Labeling Cerebral Blood Flow Images,” Journal of Cerebral Blood Flow and Metabolism 37 (2017): 3184–3192. 28058975 10.1177/0271678X16683690 PMC5584689

[45] A. P. Fan , J. Guo , M. M. Khalighi , et al., “Long‐Delay Arterial Spin Labeling Provides More Accurate Cerebral Blood Flow Measurements in Moyamoya Patients: A Simultaneous Positron Emission Tomography/MRI Study,” Stroke; A Journal of Cerebral Circulation 48 (2017): 2441–2449. 10.1161/STROKEAHA.117.017773 PMC8006795 28765286

